# Liver transplantation-related osteoporosis and fragility fractures: pathogenic pathways, risk stratification and novel targeted therapy advances

**DOI:** 10.3389/fimmu.2026.1965784

**Published:** 2026-09-17

**Authors:** Meiqi Wang, Qi Wang

**Affiliations:** 1 Department of Hepatobiliary and Pancreatic Surgery, General Surgery Center, The First Hospital of Jilin University, Changchun, China; 2 Department of Donation Intensive Care Unit, The First Hospital of Jilin University, Changchun, China

**Keywords:** bone mineral density, fragility fracture, hepatic osteodystrophy, liver transplantation, osteoporosis, risk stratification, Wnt/β-Catenin pathway

## Abstract

Liver transplantation (LT) serves as the definitive treatment for end-stage liver disease. Yet, osteoporosis (OP) and fragility fractures remain frequently overlooked skeletal complications that may impair the long-term prognosis of recipients. This review systematically summarizes recent evidence regarding epidemiological characteristics, multifactorial pathogenic pathways, risk stratification approaches, and advances in targeted interventions for LT-related bone disorders. Skeletal damage distributes across pre-transplant waiting, early post-transplant acute and long-term chronic phases; rapid bone loss and elevated fracture risk potentially peak within the first year after LT. Pre-transplant hepatic dysfunction-induced malnutrition, endocrine disturbance, and chronic inflammation lay baseline skeletal impairment. In contrast, perioperative high-dose glucocorticoids, calcineurin inhibitor toxicity, surgical denervation, and ischemia-reperfusion injury may jointly drive substantial elevation of bone turnover in the early post-transplant stage. Long-term cortical bone repair deficiency could be attributed to persistent secondary hyperparathyroidism, post-transplant diabetes, and genetic susceptibility. Dual-energy X-ray absorptiometry remains the standard bone mineral density diagnostic tool, while vertebral computed tomography attenuation value and simplified clinical scoring systems support auxiliary risk stratification for skeletal health. Generalized nutritional and lifestyle interventions combined with anti-resorptive agents form mainstream management schemes; DKK-1/Wnt/β-catenin pathway-targeted therapy shows potential as a novel intervention direction. Pediatric LT recipients feature unique bone maturation characteristics that require age-specific evaluation criteria, though unified standardized protocols remain insufficient. Current clinical practice suffers from inadequate bone health screening and pharmacotherapy coverage. Further prospective trials are warranted to validate optimized stratified screening algorithms and individualized targeted regimens for high-risk LT populations.

## 1 Introduction

Liver transplantation (LT) represents the only curative therapeutic strategy for patients with end-stage liver disease (ESLD), including chronic liver failure, acute decompensated cirrhosis, and hepatocellular carcinoma (1, 2). In recent years, remarkable breakthroughs have been achieved in LT surgical techniques, ex vivo machine perfusion preservation of donor livers, and individualized immunosuppressive regimens, substantially improving long-term survival outcomes for both allografts and recipients (3). The widespread clinical adoption of machine perfusion has expanded the donor liver pool and facilitated the large-scale utilization of aged donor livers and organs from donation after circulatory death (DCD). This advancement effectively reduces the discard rate of donor grafts, shortens pre-transplant waiting time for patients, and increases the overall volume of liver transplant procedures (4). Nevertheless, prolonged exposure to immunosuppressive agents induces multiple long-term adverse events such as renal injury and metabolic disorders, which compromise long-term allograft function, impair recipients’ quality of life, and elevate the risk of all-cause mortality (3). The latest clinical guidelines jointly issued by the American Association for the Study of Liver Diseases and the American Society of Transplantation (AASLD/AST) have established standardized diagnostic and therapeutic workflows for allograft-related complications, including post-transplant vascular injuries, biliary tract lesions, various rejection episodes, and primary disease recurrence (3).

However, osteoporosis (OP) and fragility fractures remain highly prevalent, frequently overlooked skeletal-related adverse outcomes following LT (5, 6). As the most severe clinical endpoint of OP, fragility fractures predominantly involve weight-bearing bones such as the vertebrae, hip, forearm, and pelvis (7). Following fragility fracture onset, patients face markedly elevated risks of disability and long-term all-cause mortality, accompanied by substantial medical expenditure and social care burdens (6, 7). Epidemiological statistics indicate that over 200 million individuals worldwide suffer from OP, with an overall population prevalence of approximately 18.3% (8). OP is not merely an age-related degenerative disorder; it can be secondary to a broad spectrum of systemic conditions including chronic inflammation, endocrine dysfunction, gastrointestinal malabsorption, long-term glucocorticoid administration, and solid organ transplantation, rendering it a multisystem comorbidity (9). Patients with chronic liver disease (CLD) constitute an extremely high-risk population for OP, with roughly 75% presenting concurrent OP and/or fragility fractures. This spectrum of liver-induced skeletal pathology is collectively termed hepatic osteodystrophy (HOD) (10). Accordingly, pre-transplant candidates already exhibit reduced bone mineral density (BMD) during the waiting period, laying a pathological foundation for subsequent skeletal deterioration. Skeletal disorders persist at high incidence post-transplant, and concomitant OP and fragility fractures independently increase recipients’ disability rate, cumulative complication risk, and long-term mortality (11). Multiple cohort studies have verified that most LT candidates exhibit abnormal bone mass at baseline, and the first year after transplantation constitutes a critical window of rapid bone loss, during which the risks of OP and fragility fractures rise sharply (12–14). Skeletal damage spans the entire clinical trajectory, covering the pre-transplant waiting phase, early acute post-transplant stage, and long-term follow-up, acting as a key comorbidity limiting the long-term prognosis of liver transplant recipients. Against this clinical backdrop, establishing standardized peri-transplant and long-term post-transplant skeletal health assessment protocols to identify high-risk subgroups and deliver early preventive interventions carries profound clinical significance.

## 2 Search strategy and study selection protocol

We performed a comprehensive literature search to identify relevant publications focusing on bone health complications following liver transplantation. The literature was retrieved from PubMed and Web of Science Core Collection databases. The search period spanned from database inception to the submission date of the present manuscript, with no restriction on publication year. Search terms combined Medical Subject Headings (MeSH) and free-text synonyms, including liver transplantation, end-stage liver disease, hepatic osteodystrophy, bone mineral density, fragility fracture, glucocorticoid-induced bone injury, calcineurin inhibitors, bone turnover, peri-transplant bone loss, fracture risk, and Wnt/β-catenin-related bone-targeted therapies. The search scope covered epidemiological observational studies, basic mechanistic investigations, clinical diagnostic studies, therapeutic research, authoritative reviews and clinical guidelines relevant to this topic.

Literature was screened manually based on title and abstract relevance. Potentially eligible articles were further assessed by full-text reading to judge their suitability for inclusion in this mini-review. Duplicate records were removed. Conference abstracts without sufficient detailed data, pure case reports, and studies without bone-related primary endpoints were excluded. We prioritized high-quality cohort studies, clinical trials, mechanistic research, and consensus guidelines to synthesize current evidence, summarize existing contradictions and knowledge gaps, and elaborate the pathogenesis, clinical assessment, and management strategies of bone disorders in liver transplant candidates and recipients.

## 3 Epidemiology and risk profiles of skeletal complications in LT candidates and recipients

Skeletal disorders after LT occur along two distinct temporal phases: baseline bone disease in pre-transplant ESLD candidates and progressive bone loss during the post-transplant period. Accumulated evidence from cross-sectional, retrospective, and large-scale longitudinal cohort studies consistently demonstrates a high prevalence of OP and fragility fractures among both LT candidates and long-term LT recipients (**Table 1**). Significant heterogeneity exists between these two populations regarding the prevalence of skeletal impairment, dynamic patterns of BMD decline, and independent risk factors for adverse bone outcomes. Notably, bone loss and vertebral fractures are frequently asymptomatic and occult, leading to frequent missed diagnoses under routine clinical screening protocols.

**Table 1:** Summary of core epidemiological studies on OP and fragility fractures associated with LT.

Authors (year/country)	Study type	Sample size	Core prevalence data	Core effect size and p-value	Main conclusions	Reference
Sokhi et al. (2004/USA)	Retrospective pre-LT cross-sectional study	N=104 CTP B/C cirrhosis waiting for primary LT	1. Total osteopenia 34.6%, osteoporosis 11.5% 2. Female osteopenia 38%, OP 16%; postmenopausal women OP 20% 3. CTP C patients’ BMD significantly lower than CTP B	1. Male vs female lumbar BMD P = 0.001, femoral neck P<0.0001 2. Same gender CTP C vs B at all bone sites P<0.001	Females carry a higher pre-transplant bone disease burden; liver disease severity (CTP stage) is the only independent factor driving bone loss.	(15)
Chen et al. (2024/China)	Retrospective hepatic fibrosis cross-sectional cohort	N=164 patients with F0-F1/F2/F3-F4 fibrosis	1. Liver stiffness: F0-F1 = 5.59kPa, F2 = 7.43kPa, F3-F4 = 15.48kPa 2. F2 group: all spine/hip BMD higher than F0-F1 & F3-F4 3. Correlation: F0-F1→F2 positive; F2→F3-F4 negative	1. Ordered logistic age OR = 2.047(P = 0.016), hip BMD OR = 176.368(P = 0.040) 2. F2 vs F3-F4 independent association OR = 42.198(P = 0.030) 3. All multi-site inter-group BMD P<0.05	Hepatic fibrosis has stage-dependent dual effects on bone: mild-moderate fibrosis triggers skeletal compensation, and advanced cirrhosis impairs calcium/vitamin D metabolism and accelerates bone loss.	(7)
Wibaux et al. (2011/France)	Prospective pre-LT cross-sectional study	N=99 end-stage cirrhosis waiting for LT	1. Vertebral fracture prevalence: 36.4%; 72.2% had multiple fractures 2. OP 38.4%, osteopenia 35.3%; vitamin D deficiency 87.8% 3. All patients had abnormal bone turnover markers	1. Per 1 SD BMD drop, hip fracture OR = 2.08(P = 0.001) 2. Hip BMD and alcohol abuse are independent predictors of vertebral fracture 3. MELD is negatively correlated with hip BMD (r=-0.242, P = 0.0298)	Pre-transplant patients present severe hepatic osteodystrophy with extremely high vertebral fracture and vitamin deficiency rates; hip BMD is the strongest fracture predictor.	(16)
Naseri et al. (2026/Iran)	Prospective pre-LT survival cohort	N=702 ESLD transplant candidates	1. Any-site OP 43.4%; osteopenia 36.9% 2. AIH subgroup OP highest (53.61%)	1. Multivariate Cox OP HR = 1.90(P = 0.008); PSC protective HR = 0.42(P = 0.033) 2. Kaplan-Meier log-rank P = 0.004, osteoporotic patients poorer survival	Pre-transplant osteoporosis independently increases waiting-list mortality; autoimmune hepatitis patients bear the highest skeletal damage burden.	(17)
Segal et al. (2003/Israel)	Cross-sectional long-term post-LT observational study	N=29 post-LT patients, follow-up 2–12 years	1. Any site abnormal BMD 65.5%; lumbar OP 21.4%, femoral neck OP 17.2% 2. Vitamin D deficiency 65.5%; total fracture incidence 41.4% 3. 53% fractures within the first post-LT year	1. Femoral neck BMD negatively correlated with steroid duration (r=-0.406, P = 0.029) 2. Tacrolimus was superior to cyclosporine for bone protection (P = 0.03) 3. Steroid duration is the sole independent risk factor for low femoral BMD (P = 0.017)	Long-term post-transplant patients suffer severe bone loss and vitamin D depletion; prolonged steroid therapy aggravates femoral cortical bone injury.	(18)
Chiu et al. (2019/China)	Nationwide matched post-LT hip fracture cohort	LT N = 2201, 1:10 age-sex matched controls N = 22010	1. Post-LT hip fracture rate: 0.77% vs control: 0.32% 2. Incidence rate: LT = 21.49 per 10,000 PY; control=7.52/10,000 PY 3. All hip fracture patients had long pre-LT steroid exposure	1. Multivariate Cox HR = 2.71 (95%CI 1.21–6.05, P<0.0001) 2. Recipient ≥65 vs <45 HR = 14.64 (P<0.05)	LT independently elevates long-term hip fracture risk; advanced age and chronic pre-transplant steroid use are core high-risk predictors.	(19)
Chang et al. (2023/China)	Nationwide retrospective population post-LT cohort	N=4821 long-term LT recipients	1. Overall post-LT fracture incidence: 8.7% (419/4821) 2. 24.1% of fractures occurred within the first post-LT year	1. Multivariate Cox HR: age≥65 = 1.566(P = 0.008); female=1.648 (P<0.001); pre-LT fracture=3.664 (P<0.001) 2. Prednisolone>9.18mg/d HR = 13.334(P<0.001)	Female, elderly, pre-transplant fracture, HCV, and high steroid dosage independently increase post-LT fracture risk.	(13)
Monegal et al. (2025/Spain)	Retrospective 7-year multidisciplinary protocol post-LT cohort	N=220 LT recipients with 1-year standardized follow-up 220	1. Pre-LT prevalent fragility fracture: 24%; combined OP diagnosis: 37% 2. 37% of patients had 25OHD < 10 ng/mL 3. First-year post-LT incident fracture rate 10.9% 4. Only 5% received anti-OP therapy pre-LT	1. Multivariate logistic independent risks: age>50 OR = 3.71 (P = 0.01); postmenopausal female OR = 10.0 (P = 0.005); prior fracture OR = 5.2 (P<0.001) 2. Predictive score AUC = 0.82, sensitivity 80%, specificity 72%	Even under standardized multidisciplinary bone management, ~11% of patients develop fractures within 1 year post-LT. Age, menopause, and prior fracture form a simple, high-performance risk-scoring tool; pre-transplant anti-OP intervention remains underutilized.	(20)
Li et al. (2021/Singapore)	Retrospective 10-year longitudinal post-LT cohort	N=83 LT recipients, median follow-up 80 months	1. Pre-LT OP 18.1%; post-LT OP 34.3% (P = 0.021) 2. *De novo* OP incidence 18.2%; 27.2% of patients had worsening BMD status 3. Lumbar BMD + 12% at year 10; femoral neck & total hip persistently below baseline 4. Pre-LT 90% vitamin D insufficiency	1. Lumbar BMD was significantly higher at 1y post-LT (P = 0.002) 2. Femoral neck & total hip significantly decreased at 6m (both P = 0.003)	Post-transplant OP prevalence rises significantly. Lumbar trabecular bone improves continuously within 10 years under a standard short steroid + single IV bisphosphonate regimen, while cortical bone at the hip cannot recover to pre-transplant levels. Female recipients tend to have a higher risk of progressive bone loss.	(12)
Dag et al. (2026/Turkey)	Retrospective longitudinal CT-based post-LT observational cohort	N=99 HBV-related LT recipients (59 HBV alone; 40 HBV+HCC)	1. Vertebral trabecular attenuation (L1-4) markedly decreased at postoperative 180 days, with only partial recovery at postoperative 365 days, never returning to pre-transplant baseline 2. Psoas muscle derived indices (PMA, PMI) also declined synchronously within the first 6 months post-LT 3. Baseline L1-4 attenuation, PMA and PMI showed no significant differences between HBV-alone and HBV+HCC subgroups	Time-dependent change of L1-4 attenuation: P<0.001, partial η²=0.443; pairwise PreLT vs POD180, PreLT vs POD365, both P<0.001	Synchronous bone and skeletal muscle deterioration predominantly occurs within the first six months after LT, with incomplete recovery by 12 months. After multivariable adjustment, longitudinal musculoskeletal trajectories are comparable between recipients with and without HCC, indicating early post-transplant musculoskeletal injury is mainly driven by transplant-related factors rather than underlying malignancy.	(21)

LT, liver transplantation; pre-LT, pre-liver transplantation; post-LT, post-liver transplantation; VF, vertebral fragility fracture; HF, hip fragility fracture; BMD, bone mineral density; OP, osteoporosis; PY, person-year; CTP, Child–Pugh score; MELD, Model for End-Stage Liver Disease; AIH, autoimmune hepatitis; PSC, primary sclerosing cholangitis; Tac, tacrolimus; CsA, cyclosporine A; 25OHD, 25-hydroxyvitamin D; PMA, psoas muscle area; PMI, psoas muscle index.

### 3.1 Pre-transplant skeletal complications: prevalence and fracture risks in ESLD candidates

Multiple clinical studies have confirmed a markedly elevated risk of OP and subsequent fragility fractures among LT candidates (14). A cross-sectional study conducted by Sokhi et al. in 2004, enrolling 104 patients with cirrhosis, revealed that BMD declined progressively with the worsening severity of cirrhosis. Significant disparities in BMD were also identified across genders and between patients with Child–Turcotte–Pugh grade B and C cirrhosis (15). A retrospective cohort study by Chen et al. further validated the correlation between hepatic fibrosis and skeletal damage. The study stratified 164 liver disease patients into F0–F1, F2, and F3–F4 fibrosis groups via two-dimensional shear wave elastography, and detected statistically significant differences in multi-site BMD across the three groups (P < 0.05). Patients with F2 fibrosis exhibited substantially higher lumbar and hip BMD values compared to those with mild or severe fibrosis. As fibrosis advanced from F2 to F3–F4, liver stiffness was negatively correlated with BMD. After adjusting for confounding variables, reduced total hip BMD was identified as an independent correlate of hepatic fibrosis (OR = 176.368, P = 0.040) (7).

A prospective study by Wibaux et al., involving 99 ESLD transplant candidates, reported a vertebral fracture prevalence of 36%, OP prevalence of 38%, and osteopenia prevalence of 35%. Decreased BMD was significantly associated with vertebral fracture risk: each 1-standard-deviation reduction in BMD corresponded to odds ratios of 1.45 for spinal fractures, 2.1 for total hip fractures, and 2.0 for femoral neck fractures (all P < 0.05) (16). Consistent with these findings, a prospective cross-sectional study by Naseri et al., including 702 LT candidates, found OP in 43.4% and osteopenia in 36.9% of participants, while merely 19.7% maintained normal BMD. Multivariate adjustment confirmed OP as an independent risk factor for all-cause mortality during the waiting period (HR = 1.90, P = 0.008), and Kaplan–Meier survival curves demonstrated significantly lower cumulative survival in patients complicated with OP (17). Collectively, these data indicate that pre-transplant candidates commonly present with asymptomatic vertebral fractures and osteopenia, mandating routine systematic skeletal assessment for all individuals awaiting LT.

### 3.2 Longitudinal trends of post-transplant bone loss and *de novo* fragility fractures

Longitudinal cohort studies uniformly identify the first 12 months after LT as a high-risk window for rapid bone loss and incident fragility fractures. Pre-transplant glucocorticoid(GC) exposure, prior fracture history, and sustained post-operative administration of GCs and immunosuppressants all exacerbate the risk of skeletal injury. Segal et al. performed a long-term follow-up study with a 2–12 year observation period enrolling 29 LT recipients, in which 65.5% of subjects were found to have osteopenia. The study defined post-operative vitamin D deficiency, long-term cyclosporine use, and duration of GC therapy as three independent risk factors for bone damage. However, no correlation was detected between total cumulative GC dosage and skeletal lesions (18). Chiu et al. conducted a large retrospective analysis based on Taiwan’s National Health Insurance Research Database (NHIRD) covering 2,201 LT recipients. The incidence density of hip fractures after LT was significantly higher than that in the general population (HR = 2.71, P < 0.0001). All patients who sustained hip fractures had received GCs for more than 30 days pre-transplant (19), demonstrating that GC exposure both before and after transplantation elevates the risk of adverse skeletal events.

A cohort study by Chang et al., also constructed from NHIRD data and comprising 4,821 LT recipients, reported an overall post-transplant fracture incidence of 8.7%, with 24.1% of all fracture episodes occurring within the first year post-operatively. Multivariate adjustment identified age ≥65 years at transplantation, female sex, fracture history within one year before LT, hepatitis C virus infection, and alcohol consumption as independent risk factors for post-transplant fractures. Daily prednisone dosage exhibited a dose-dependent association with fracture risk; the odds ratio reached 13.334 for patients receiving daily prednisone doses exceeding 9.18 mg (P < 0.001) (13). A retrospective study by Monegal et al. enrolling 220 LT patients yielded comparable results: 24% of subjects presented with fragility fractures pre-transplant, and 77% suffered from either osteopenia or OP. Even with standardized bone-protective interventions throughout follow-up, 10.9% of patients developed new fragility fractures within the first post-operative year. After adjusting for confounders, age > 50 years, post-menopausal status, and previous fracture history were confirmed as independent predictors of fracture occurrence (20). Furthermore, Li et al. conducted a 10-year stratified longitudinal follow-up of 83 LT recipients. The baseline prevalence of OP was 18.1%, which rose significantly to 34.3% after transplantation (P = 0.021). Marked site-specific heterogeneity was observed in BMD recovery: lumbar BMD increased by 12% relative to baseline at the 10-year mark, whereas femoral neck and total hip BMD never returned to pre-transplant levels (12).

Synthesizing these longitudinal follow-up data, the risk of rapid bone loss and *de novo* fractures surges in the early post-transplant phase, and skeletal damage persists chronically with divergent regenerative capacities across distinct bone sites. Clinicians are advised to strengthen skeletal surveillance and early intervention within the high-risk post-operative window, alongside establishing long-term skeletal follow-up protocols for high-risk recipients, including older people, women, and individuals with a history of GC exposure.

Synthesizing these longitudinal follow-up data, the risk of rapid bone loss and *de novo* fractures surges in the early post-transplant phase, and skeletal damage persists chronically with divergent regenerative capacities across distinct bone sites. Recent longitudinal opportunistic-CT-based evidence further depicts synchronized musculoskeletal alterations after liver transplantation among hepatitis B-related recipients, irrespective of concurrent hepatocellular carcinoma status (21). In a retrospective cohort with serial CT assessments, both vertebral trabecular attenuation and psoas muscle indices declined prominently within the first six postoperative months, exhibiting only partial recovery by 12 months without returning to pre-transplant baseline levels. Of note, the longitudinal trajectories of bone- and muscle-related CT-derived parameters were comparable between recipients with and without hepatocellular carcinoma after multivariable adjustment, implying that early post-transplant musculoskeletal deterioration is predominantly driven by transplantation-associated insults rather than underlying malignancy status (21). Clinicians are advised to strengthen skeletal surveillance and early intervention within the high-risk postoperative window, alongside establishing long-term skeletal follow-up protocols for high-risk recipients, including older people, women, and individuals with a history of GC exposure.

## 4 Pathogenetic mechanisms of LT-associated skeletal complications

The pathogenesis of OP and fragility fractures after LT exhibits multifactorial synergistic effects and temporally stratified characteristics, spanning three distinct clinical phases: the pre-transplant waiting period, the early acute post-transplant phase, and long-term late follow-up. Pre-existing HOD induced by ESLD serves as the fundamental predisposing basis for skeletal deterioration. Multiple perioperative insults, including immunosuppressive agents, surgical stress, and hepatic denervation, may collectively drive rapid bone loss in the early post-transplant stage. In contrast, persistent genetic susceptibility, chronic metabolic disturbances, and dysregulated bone signaling pathways potentially mediate progressive cortical bone changes with limited reparative potential during long-term post-transplant surveillance (**Figure 1**).

**Figure 1:**
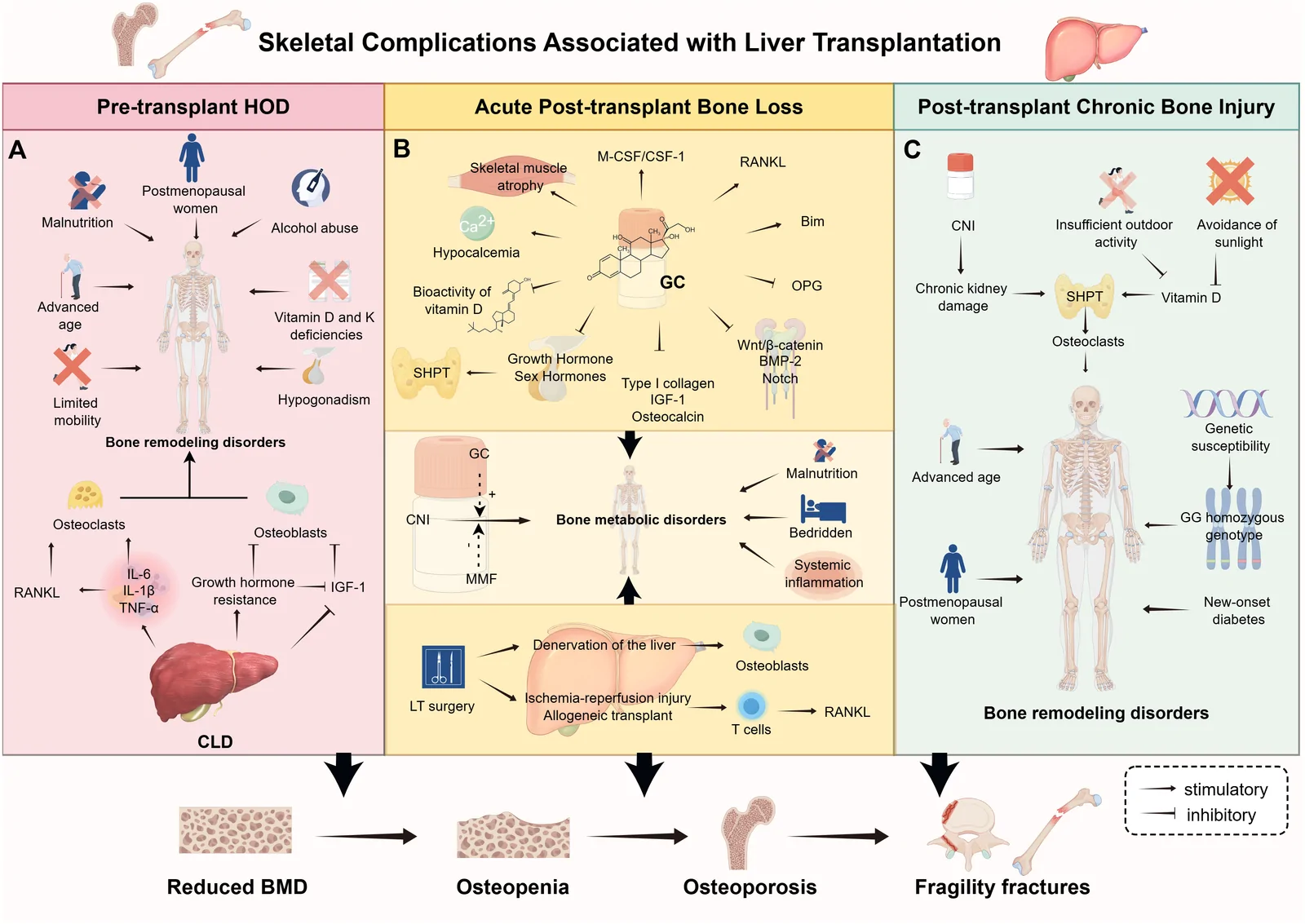
Temporally stratified pathogenic cascades underlying skeletal complications after LT, divided into three clinical phases. **(A)** Pre-transplant HOD: multiple risk factors related to CLD disrupt bone remodeling via inflammatory cytokines, insufficient IGF-1 secretion, and hypogonadism, leading to reduced BMD. **(B)** Acute post-transplant bone loss (0–6 months): GC and CNI synergistically induce high-turnover bone metabolism through dysregulating RANKL/OPG balance and suppressing osteogenic pathways; surgical hepatic denervation and ischemia-reperfusion injury further exacerbate bone loss, progressing to osteopenia and osteoporosis. MMF alleviates CNI-mediated bone toxicity. **(C)** Chronic long-term bone injury (>6 months): persistent SHPT, genetic susceptibility, insufficient vitamin D, and post-transplant diabetes drive progressive cortical bone deterioration with limited reparative potential. All three phases sequentially aggravate skeletal lesions and eventually cause OP and fragility fractures. LT, liver transplantation; HOD, hepatic osteodystrophy; CLD, chronic liver disease; BMD, bone mineral density; GC, glucocorticoids; CNI, calcineurin inhibitors; SHPT, secondary hyperparathyroidism.

### 4.1 Pre-transplant HOD: pathogenic drivers in ESLD patients

The pathogenesis of LT-related OP involves a combination of pre-existing baseline skeletal lesions and post-transplant secondary stimuli. Multiple studies have delineated the age-dependent trajectory of bone mass: BMD peaks at approximately 40 years of age, maintains calcium metabolic homeostasis from 40 to 50 years old, and thereafter undergoes continuous decline. Individuals aged 50–60 years are prone to osteopenia, which may gradually progress to OP, accompanied by progressive depletion of systemic vitamin D reserves (7). Beyond advanced age itself, multiple adverse conditions including inadequate nutritional intake, alcoholic liver disease, chronic cholestatic liver disorders, vitamin D deficiency, hypogonadism and prolonged immobilization collectively trigger pre-transplant HOD (12, 22). A history of pre-transplant vertebral fractures or reduced BMD, as well as prior GC exposure, further elevates the long-term risk of fragility fractures (22).

Among patients with alcoholic liver disease, excessive alcohol directly suppresses osteoblastic activity and reduces serum osteocalcin levels. Alcohol intake has been validated as an independent risk factor for OP, conferring a 3-fold higher fracture risk relative to the general population (23). For patients with chronic cholestatic liver disease, chronic accumulation of bile acids and bilirubin impairs osteoblastic function and disrupts intestinal absorption of fat-soluble vitamins D and K, thereby disturbing physiological bone remodeling (5). The severity of hepatic injury and cholestasis is positively correlated with the magnitude of bone loss (13).

Dysregulated sex hormone homeostasis constitutes a core mediator of disturbed bone metabolism. In CLD patients, hepatic estrogen clearance is impaired while peripheral androgen-to-estrogen conversion is enhanced, leading to relative hyperestrogenemia. Elevated estrogen negatively feeds back on the hypothalamic–pituitary–gonadal axis to reduce secretion of gonadotropins and adrenocorticotropic hormone, ultimately resulting in hypogonadism and diminished circulating androgens. Circulating levels of both estrogens and androgens are markedly lower than in healthy controls, failing to inhibit osteoclast activation adequately and consequently causing insufficient bone formation and excessive bone resorption (24). The rate of bone loss is further accelerated in postmenopausal women with CLD, presenting more severe OP phenotypes (25).

Apart from endocrine disruption, sustained hepatocellular injury in CLD drives chronic low-grade inflammation, whereby immune cells secrete abundant IL-6, IL-1β and TNF-α. These pro-inflammatory cytokines directly modulate osteoclast proliferation and function, and indirectly amplify osteoclastic activity by upregulating receptor activator of nuclear factor κB ligand (RANKL) expression in osteoblasts (24, 25). A single-center retrospective longitudinal cohort conducted by Huldén et al. enrolling 102 patients with cirrhosis of heterogeneous etiologies demonstrated that higher baseline pre-transplant TNF-α (r=-0.47, P = 0.012) and serum cortisol (r=-0.49, P = 0.008) were correlated with more prominent declines in hip BMD after LT (26). CLD is also frequently complicated with growth hormone resistance and reduced hepatic synthesis of insulin-like growth factor-1 (IGF-1), which impairs osteogenic signaling cascades (5). As a central osteogenic mediator predominantly synthesized by the liver under growth hormone stimulation, IGF-1 production declines with progressive hepatic synthetic dysfunction, directly suppressing bone formation (24).

Collectively, ESLD induces pre-transplant HOD via synergistic pathways encompassing malnutrition, alcohol toxicity, cholestasis, endocrine imbalance, chronic inflammation, and impaired growth factor synthesis, laying a pathological foundation for rapid bone loss following transplantation.

### 4.2 Acute bone loss in the early post-transplant phase: key pathogenic mediators

Theoretically, restoration of hepatic synthetic function after LT normalizes hepatic production of vitamin D transport proteins, relieves toxic free bile acid accumulation, and improves appetite and intestinal nutrient absorption, which should theoretically restore adequate skeletal nutrient supply (27). Nevertheless, abundant clinical evidence indicates elevated risks of bone loss and fragility fractures post-transplant. Patients typically exhibit a low-turnover bone metabolic state prior to LT, which shifts to drastically elevated bone turnover within 3–6 months after surgery, triggering acute rapid bone loss (25, 28–31). Recipients with baseline OP demonstrate further elevated risks of post-transplant fragility fractures (32). Early post-operative malnutrition, prolonged bed rest and perioperative systemic inflammation exacerbate bone metabolic derangements (33). GCs and other immunosuppressants represent the core modifiable risk factors driving hyperactive bone turnover and excessive bone resorption, substantially increasing recipients’ disability risk, complication burden and all-cause mortality (11).

GCs serve as cornerstone immunosuppressive agents after LT, administered at high initial doses followed by rapid tapering; supplementary high-dose GC therapy is required during episodes of acute rejection (34). All LT recipients receiving maintenance GC therapy exhibit elevated risks of skeletal complications (35). Early rapid bone loss is primarily attributed to perioperative high-dose GC exposure (36). A 2000 study by Trautwein et al. reported significantly elevated serum C-telopeptide (a bone resorption marker) in patients receiving daily prednisone >7.5 mg, with bone resorption activity rising linearly alongside increasing GC dosage (33). Even with subsequent GC tapering, early bone loss may already be established, substantially increasing the risk of vertebral and hip fragility fractures (30).

GCs exert dual direct and indirect effects on osteoblasts, osteoclasts and osteocytes to concurrently suppress bone formation, accelerate bone resorption and induce osteonecrosis (29). At the cellular level, GCs upregulate RANKL and downregulate osteoprotegerin (OPG), disrupting the physiological RANKL/OPG ratio (30, 34, 36). They also increase macrophage colony-stimulating factor (M-CSF/CSF-1), an essential cytokine supporting survival and activation of osteoclast precursors, synergistically promoting osteoclast proliferation and differentiation to amplify bone resorption (30, 36). Meanwhile, GCs inhibit canonical osteogenic signaling cascades including Wnt/β-catenin, BMP-2 and Notch, blocking directed differentiation of osteoprogenitor cells (30, 34, 36). GCs further reduce synthesis of type I collagen, IGF-1 and osteocalcin, while upregulating pro-apoptotic protein Bim to induce apoptosis of osteoblasts and osteocytes, ultimately disrupting bone remodeling homeostasis (27, 30, 36, 37). Systemically, GCs diminish vitamin D bioavailability, impair intestinal calcium absorption and augment urinary calcium excretion to induce hypocalcemia (30, 34, 36, 38). They also suppress secretion of growth hormone, IGF-1 and endogenous sex hormones, triggering secondary hyperparathyroidism and further perturbing bone remodeling equilibrium (30, 34). GC-induced myasthenia and reduced physical activity additionally provoke skeletal muscle atrophy, aggravating disuse bone loss (30, 34).

Beyond GCs, cyclosporine (CsA) and tacrolimus (TAC), two calcineurin inhibitors (CNIs), constitute first-line immunosuppressants for LT recipients. They exert immunosuppressive effects by inhibiting calcineurin activity and blocking synthesis and secretion of T-cell-derived inflammatory cytokines (34). Both agents disrupt bone metabolism and exacerbate post-transplant bone loss, with cyclosporine exerting stronger pro-resorptive effects (30). Clinically, CNIs are frequently co-administered with GCs, producing synergistic skeletal toxic effects that markedly increase the incidence of bone complications. Mycophenolate mofetil (MMF) can be combined with CNIs to reduce CNI exposure and mitigate skeletal toxicity (39).

Apart from pharmaceutical toxicities, surgical trauma contributes to acute bone loss, with hepatic denervation representing a potential unique mechanistic factor. Kissler et al. established a rat orthotopic LT animal model. They indicated that surgical hepatic denervation independently reduces lumbar and femoral BMD, pathologically manifested as impaired osteoblastic function and attenuated trabecular thickness (40). Such neural injury might partially explain the clinical phenomenon of sustained bone loss and recurrent fragility fractures in patients years after GC discontinuation. Fábrega et al. observed synchronous significant elevations in circulating OPG and RANKL within 14 days post-transplant. They hypothesized that intraoperative ischemia-reperfusion injury and allogeneic graft stimulation may activate systemic T lymphocytes to release abundant inflammatory cytokines and RANKL. Endothelial cells compensate by upregulating OPG secretion to counteract excessive bone resorption. Yet, the compensatory OPG response remains insufficient to fully abrogate rapid early bone loss, which may align with the predominant high-turnover bone metabolic phenotype after LT (41).

### 4.3 Chronic skeletal injury in the late post-transplant phase: persistent dysregulated signaling pathways

Six months after transplantation marks the onset of the long-term follow-up phase. Gradual hepatic functional recovery and tapering GC regimens partially alleviate excessive bone turnover (25). Adult bone tissue consists of 80% cortical bone and 20% trabecular bone. Distinct skeletal sites exhibit divergent proportions of these two compartments, resulting in heterogeneous bone remodeling rates (37). The lumbar spine is predominantly trabecular bone (66%) with high metabolic activity, enabling rapid recovery following hepatic recovery. In contrast, the femoral neck contains over 75% cortical bone, exhibiting slow and incomplete tissue repair (42). These site-specific differences between trabecular-predominant and cortical-predominant bone compartments help interpret divergent longitudinal findings from DXA monitoring in LT cohorts, as observed in A 3-year follow-up study by Monegal et al., enrolling 45 LT recipients, documented significant declines in lumbar and femoral neck BMD from 3 to 6 months post-operatively. Lumbar BMD recovered to baseline levels by year 2, while femoral neck BMD achieved only partial restoration and did not fully recover within 3 years (43). As can be seen from this, Early reductions were most pronounced within trabecular-rich lumbar regions. In contrast, femoral cortical-dominant sites showed limited capacity for full restoration even at later time-points. Long-term CNI-induced chronic nephropathy triggers persistent secondary hyperparathyroidism, which suppresses bone remodeling and partially accounts for the delayed repair of cortical bone relative to trabecular bone (23, 31). Insufficient outdoor activity and chronic sun avoidance post-transplant maintain sustained vitamin D deficiency, further inducing secondary hyperparathyroidism, continuously activating osteoclasts and driving progressive cortical bone deterioration with limited capacity for recovery (18).

Elderly recipients and postmenopausal women represent high-risk subgroups for late-onset OP and fragility fractures. Genetic susceptibility is another non-negligible contributor to chronic skeletal damage. A retrospective clinical study by Yanagawa et al. involving 253 LT recipients demonstrated significantly higher OP prevalence in carriers of the GG homozygous genotype at the vitamin D receptor BsmI locus (P = 0.021) (38). New-onset diabetes mellitus after LT also chronically disrupts bone homeostasis. A real-world retrospective cohort by Bégin et al. enrolling 155 LT recipients identified post-transplant diabetes as an independent risk factor for long-term fragility fractures (44). Insulin resistance, accumulation of advanced glycation end products, chronic low-grade inflammation, and oxidative stress synergistically disrupt physiological bone remodeling (45).

## 5 Stratified diagnosis and multidisciplinary management of skeletal complications in liver transplant candidates and recipients

OP is not merely an isolated comorbidity but a dynamic surrogate marker reflecting overall disease severity, physical frailty, and immune dysfunction in patients with ESLD receiving LT. Accordingly, integrated, multi-layered and multidisciplinary care protocols are warranted for both pre- and post-transplant LT patients to mitigate skeletal adverse outcomes (17). Nevertheless, substantial gaps remain in real-world clinical implementation of bone health surveillance and intervention. Clinical epidemiological data demonstrate that over 30% of LT candidates are diagnosed with OP before transplantation; however, only 11% receive bone-protective pharmacotherapy pre-transplant, with a modest increase to merely 18% in the post-transplant setting. Additionally, merely 34.5% of recipients undergo follow-up BMD testing after surgery, leaving a large proportion of patients with osteopenia without timely preventive intervention (11).

### 5.1 Standardized clinical diagnostic system for transplant-associated skeletal complications

#### 5.1.1 Stratified screening strategies and diagnostic modalities for bone mass assessment

Dual-energy X-ray absorptiometry (DXA) is the globally recognized gold standard for BMD measurement, with T-scores serving as the core diagnostic index for OP (24). A T-score denotes the standard deviation difference between an individual’s BMD and the peak bone mass of age-matched healthy individuals of the same sex. Per the World Health Organization (WHO) diagnostic cut-offs: T ≤ −2.5 defines OP, −2.5 < T < −1.0 indicates osteopenia, and T > −1.0 represents normal bone mass (46). Pre-transplant baseline DXA screening combined with serial post-transplant reassessment enables early intervention and reduces the incidence of peri-transplant OP and fragility fractures. Despite its diagnostic superiority, DXA suffers from limited accessibility due to equipment shortages and separate appointment requirements, rendering frequent routine follow-up impractical for transplant recipients (5). Excessively frequent screening imposes economic burdens and consumes medical resources, whereas insufficient surveillance delays early intervention and elevates fracture-related healthcare expenditures (47). Consensus on optimal post-transplant DXA intervals remains absent across guidelines and clinical studies. The joint 2012 clinical guidelines issued by the American Association for the Study of Liver Diseases and American Society of Transplantation (AASLD/AST) proposed stratified follow-up schedules: recipients with normal pre-transplant BMD undergo DXA scanning every 2–3 years within the first five post-transplant years; patients with pre-existing osteopenia receive annual scans over the same period; surveillance intervals beyond 5 years are adjusted based on longitudinal BMD trends and concomitant comorbidities (48). Díaz et al. recently recommended lumbar plus bilateral hip DXA at 6 months post-transplant, with annual monitoring for osteopenia and 2–3-year intervals for OP patients (49). A large single-center retrospective cohort from the Mayo Clinic enrolling 402 LT recipients aged over 50 years established age-specific follow-up protocols: patients with baseline normal bone mass receive their first DXA at 15 months post-transplant and a second scan at 4–5 years; those on bisphosphonate therapy for pre-transplant osteopenia only require DXA at 2 years without annual surveillance (47).

Beyond resource constraints, DXA has inherent analytical limitations (**Table 2**). Rodríguez-Aguilar et al. emphasized that complete interpretation of DXA reports requires evaluation of lumbar, femoral neck, trochanteric, and Ward’s triangle measurements rather than relying solely on T-scores. BMD only quantifies bone mineral content and cannot detect microstructural or matrix defects, which frequently coexist with mineral loss in patients with primary biliary cholangitis (23). DXA fails to distinguish cortical from trabecular bone and exhibits limited sensitivity for fracture prediction, as aortic calcification, osteophytosis, and degenerative joint disease interfere with readings (50). Ascites causes falsely reduced lumbar BMD values, while spinal degenerative lesions artificially elevate measured bone density (57). Given these drawbacks and limited clinical uptake of DXA, cost-effective, low-radiation auxiliary screening tools are urgently required (53). Multiple cohort studies support the additive value of spinal radiography for detecting occult vertebral fractures. Krol et al.’s cohort of 164 transplant candidates documented a 56% prevalence of pre-transplant vertebral fractures without significant correlations between lumbar/femoral neck BMD stratification and fracture occurrence, justifying routine pre-transplant lateral spinal X-ray screening to identify asymptomatic vertebral lesions (58). A cross-sectional study by Jurina et al. involving 90 waiting-list patients reported an overall spinal fragility fracture prevalence of 48.9%, with merely 22.7% of fractured patients presenting back pain; fracture events showed no association with global hip or spinal BMD, meaning DXA alone misses numerous asymptomatic fractures in patients with normal bone mass. Combined pre-transplant spinal radiography and DXA is therefore recommended (57). Zavatta et al.’s large retrospective cohort of 366 LT candidates yielded congruent results, reporting an overall fragility fracture prevalence of 42.3%, 93.5% of which were vertebral fractures, and 41.3% of patients sustaining multi-level spinal injuries, most without symptomatic manifestations (59).

**Table 2:** Summary of screening modalities for bone-health evaluation in liver-transplant recipients.

Modality	Key advantages	Main limitations	Recommended clinical application scenarios	Level of available evidence (CEBM)	Reference
DXA	Gold standard reference for BMD measurement; standardized pediatric- and adult-specific reference datasets; low radiation exposure	Cannot detect vertebral fractures directly; areal BMD is affected by body size, stature, and degenerative changes; cannot discriminate between trabecular and cortical bone compartment changes	Baseline and serial follow-up bone health assessment for adult and pediatric LT recipients; primary diagnostic tool for evaluating low bone mineral status	2a	(46, 23, 50–52)
Vertebral CT attenuation (HU values)	Derived from routine abdominal CT performed for transplant surveillance; opportunistic bone health screening without additional radiation or extra visits; good capacity for reflecting trabecular skeletal status	Not a direct measurement of BMD; cannot replace DXA for formal osteoporosis diagnosis	Auxiliary preliminary skeletal risk stratification when DXA is logistically inaccessible; serial observational evaluation within existing CT follow-up workflows	2b	(53, 50, 5, 14)
Spine radiography/VFA	VFA can be completed during DXA scanning with minimal extra radiation; valuable for identifying asymptomatic fragility fractures	Diagnostic reliability is reduced for isolated Genant grade-1 mild vertebral deformities; upper-thoracic vertebrae may be unevaluable due to anatomical overlay, scoliosis, or motion artifacts; Dedicated validation data in pediatric liver-transplant cohorts are lacking	Screening for prevalent vertebral fragility fractures, especially for recipients with high fracture risk (long-term GC exposure, known low bone mass)	2b	(31, 54)
Clinical fracture-risk scoring systems	Non-invasive, low-cost; integrates readily available clinical risk factors (age, sex, glucocorticoid exposure, prior fracture history, liver disease severity); facilitates risk-stratified clinical decision-making	Does not provide direct bone-density or imaging information; performance has not been fully validated specifically in LT populations; cannot replace imaging-based bone evaluation	Initial risk triage for transplant candidates and recipients; aid in identifying high-risk subgroups that require subsequent dedicated bone-imaging work-up	4	(55, 56, 20)

CEBM, Oxford Centre for Evidence-based Medicine 2009 diagnostic domain evidence level grading system; DXA, dual-energy X-ray absorptiometry; BMD, bone mineral density; HU, Hounsfield unit; VFA, vertebral fracture assessment; LT, liver transplantation.

Computed tomography (CT) is routinely ordered for LT recipients, enabling assessment of vertebral radiodensity for skeletal-health screening, without extra appointments, radiation exposure, or additional medical costs, making it suitable for pre-transplant baseline and long-term post-transplant skeletal monitoring (53). A retrospective Mayo Clinic study of 91 transplant candidates demonstrated that bone computed tomography (BCT) for vertebral biomechanical assessment achieves a screening sensitivity of 83.3% and negative predictive value of 91.7% relative to DXA as the reference standard, reliably ruling out patients without skeletal injury (50). A cross-sectional analysis by Nachef et al. of 376 waiting-list patients identified L1 CT attenuation value as an independent predictor of vertebral fracture: each 42-Hounsfield unit (HU) reduction in L1 radiodensity elevated fracture odds by 6.37-fold (OR = 6.37, P<0.0001). Patients with L1 CT ≤100 HU exhibited a 39% fracture incidence (OR = 7.20) and constituted an extremely high-risk pre-transplant subgroup, with an area under the curve (AUC) of 0.86 for fracture prediction using CT attenuation metrics (14). Leveraging routine CT scans for preliminary skeletal evaluation streamlines pre-transplant workups and alleviates patient burdens (50). Dag et al. proposed a tiered diagnostic algorithm: patients with markedly reduced vertebral HU values receive confirmatory DXA and early anti-osteoporotic therapy. At the same time, individuals with normal radiodensity and no fracture signs continue routine follow-up with deferred DXA. Nevertheless, vertebral HU measurement functions only as an auxiliary screening modality and cannot fully substitute DXA for definitive OP diagnosis (**Table 2**) (5).

#### 5.1.2 Risk stratification tools for fragility fracture prediction

The Fracture Risk Assessment Tool (FRAX) is a standardized predictive model for the general population that estimates 10-year probabilities of OP and hip fractures (55). Multiple simplified scoring systems tailored to liver transplant populations have been developed in recent years to streamline high-risk patient identification. Abate et al. constructed a 0–9 point skeletal risk score based on 5-year follow-up data from 264 LT recipients at the Mayo Clinic, incorporating predictors: female sex (+1), non-African ancestry (+2), pre-transplant fracture history (+1)/post-transplant *de novo* fracture (+2), one acute rejection episode (+1)/≥2 rejection episodes (+2), T-score between −2.5 and −1.0 (+1)/T ≤ −2.5 (+2). The model effectively segregates patients into low, intermediate, and high-risk subgroups; individuals scoring >5 are advised to undergo spinal imaging to screen for silent vertebral fractures and initiate early anti-resorptive therapy (56). A retrospective cohort by Monegal et al. enrolling 220 transplant recipients identified age >50 years, postmenopausal status, and prior fracture history as independent fracture predictors, forming a simplified 0–3 point scoring system with satisfactory sensitivity and specificity for rapid clinical risk stratification and intensified early intervention (20). While these simple scores are clinically convenient for rapid preliminary screening, all supporting evidence derives from retrospective analyses; their predictive performance requires prospective validation, and they should only be used as adjunct risk-assessment tools (**Table 2**).

### 5.2 Peri-transplant stratified management: universal basic skeletal interventions

Fundamental lifestyle and nutritional interventions apply to all LT candidates and recipients, featuring low implementation barriers and broad clinical applicability. Clinicians should initiate preventive anti-osteoporotic strategies early for high-risk subgroups including the elderly, postmenopausal women, and patients with prolonged pre-transplant glucocorticoid exposure (19). All peri-transplant patients are counseled to discontinue tobacco and excessive alcohol intake (32). Post-transplant dietary regimens should adhere to low-fat, low-sodium guidelines with adequate high-quality protein and dietary fiber, alongside increased consumption of calcium and vitamin D-rich foods to sustain skeletal homeostasis (27). Patients are encouraged to ambulate early after surgery and perform weight-bearing exercise 3 times weekly for 30 minutes per session (60). Supplementary nutritional support is mandatory alongside dietary modification: daily calcium supplementation of 1000–1200 mg (calcium carbonate administered with meals), and daily 800 IU vitamin D (or a single biweekly 260 μg dose), targeting serum 25(OH)D levels >30 ng/mL (23, 30, 60). Patients complicated by secondary hyperparathyroidism or chronic kidney disease may receive adjunct active vitamin D (calcitriol 0.25 μg twice daily) (23, 29, 30). Vitamin K also participates in bone remodeling by regulating osteocalcin γ-carboxylation; osteocalcin acts as a vitamin K-dependent bone formation marker, and concurrent vitamin K supplementation may attenuate post-transplant BMD loss (53).

Guidelines from the European Calcified Tissue Society recommend early bisphosphonate therapy for intermediate-to-high-risk skeletal candidates awaiting transplantation (30). Multiple clinical studies confirm anti-resorptive efficacy for alendronate, ibandronate, pamidronate and zoledronic acid, with consistent improvements in lumbar and hip BMD (29). Zoledronic acid administered for one year, or alendronate/ibandronate over two years, sustainably preserves lumbar, femoral neck and total hip bone mass (28, 61, 62) Two years of oral alendronate significantly elevates lumbar and femoral BMD in OP patients, with continuous 2–3-year therapy yielding progressive BMD gains for osteopenic individuals (63). Nevertheless, several investigations have raised concerns regarding bisphosphonate efficacy. Monegal et al. demonstrated that pamidronate alleviates lumbar and greater trochanter bone loss yet confers minimal protection against femoral cortical bone deterioration and fails to reduce overall fracture risk (64). Bégin et al.’s cohort revealed persistently elevated short-term fragility fracture rates despite standardized post-transplant risedronate or alendronate treatment, alongside irreversible femoral neck cortical bone loss (44). Furthermore, long-term oral alendronate correlates with increased radiographic jaw abnormalities and elevated osteonecrosis of the jaw risk in LT recipients with OP (65).

For patients intolerant to bisphosphonates or with concurrent renal impairment, denosumab represents an alternative therapeutic option. As a monoclonal antibody against RANKL, denosumab inhibits osteoclast differentiation, activation, and survival by blocking RANKL-receptor binding, thereby suppressing excessive bone resorption (29). A retrospective study by Brunova et al. enrolling 63 combined liver, kidney and pancreas-kidney transplant patients with OP reported favorable outcomes with subcutaneous denosumab 60 mg every six months plus long-term calcium and vitamin D supplementation. After a mean treatment duration of 1.65 years, lumbar OP prevalence declined from 74.6% to 27% and femoral OP from 54% to 36%, with the most pronounced lumbar BMD gains observed in liver transplant subgroups (66).

Recent mechanistic research targeting the Wnt/β-catenin pathway identifies potential novel therapeutic avenues for LT-associated OP. Beyond glucocorticoid-mediated Wnt/β-catenin suppression, a follow-up cohort by Kuo et al. of 25 living-donor LT recipients without anti-bone pharmacotherapy found significant post-hepatic recovery upregulation of DKK-1, a canonical Wnt pathway inhibitor. Patients developing *de novo* fractures within three years exhibited higher baseline OPG, lower RANK, elevated OPG/RANKL ratios, and increased DKK-1 concentrations (67). DKK-1 may bind osteoblastic LRP5/6 Wnt receptors to block downstream β-catenin signaling, potentially suppressing osteoprogenitor proliferation and differentiation while possibly inducing osteoblast apoptosis (68). DKK-1 additionally might downregulate OPG expression, disrupting the OPG/RANKL equilibrium and indirectly amplifying RANKL-driven osteoclastogenesis (67). Preclinical animal experiments utilizing DKK1 antisense oligonucleotides (DKK1-AS) restore osteogenic gene transcription, enhance bone mechanical properties, promote bone formation, and inhibit osteoclast generation in osteogenesis imperfecta mouse models (69). Therefore, therapeutic activation of the Wnt/β-catenin cascade or targeted DKK-1 inhibition represents promising potential novel strategies for LT-related OP (67, 68).

For recipients sustaining post-transplant vertebral fragility fractures, Sonmez et al. compared percutaneous vertebroplasty and balloon kyphoplasty. Balloon kyphoplasty achieves faster pain relief, earlier mobilization, and fewer procedural complications, facilitating accelerated rehabilitation; however, small sample sizes limit generalizability, requiring large multi-center trials to validate long-term safety and efficacy (70).

### 5.3 Individualized skeletal health management for pediatric liver transplant recipients

Most existing skeletal disorder research focuses on adult LT patients, with limited evidence specific to pediatric recipients, who undergo continuous skeletal maturation. No globally unified criteria for pediatric bone health evaluation have been established (51). Pediatric bone reference ranges differ substantially from adults, and pre-transplant screening or post-transplant pharmacotherapy may interfere with linear growth, necessitating specialized clinical attention for this unique population.

DXA bone mass assessment for children relies on Z-scores rather than adult T-scores. A Z-score quantifies the deviation of a child’s BMD from age-, sex-, and height-matched healthy peers. A Z-score ≤ −2.0 indicates low bone mineral density for chronological age (51); critically, pediatric osteoporosis cannot be diagnosed by DXA Z-score alone, per official position statements from the International Society for Clinical Densitometry (ISCD) (52). To establish a diagnosis of pediatric osteoporosis, supporting clinical evidence of fragility fractures is mandatory: this includes either one or more atraumatic vertebral fractures irrespective of Z-score, or a clinically meaningful long-bone fracture burden (≥2 long-bone fractures before 10 years of age, or ≥3 long-bone fractures by 19 years of age) in conjunction with a Z-score ≤ −2.0 (52). A Z-score between −2.0 and −1.0 represents borderline bone mass status, while a Z-score > −1.0 indicates age-appropriate normal bone mass (51). Notably, a Z-score higher than −2.0 does not completely exclude elevated skeletal fragility risk among chronically ill pediatric liver-transplant recipients (71).

A cross-sectional cohort by Pornsiripratharn et al. of 147 children with chronic liver disease demonstrated a high prevalence of vertebral fractures regardless of transplant status, most without back pain. Height Z-score < −2.0 constituted an independent vertebral fracture risk factor (OR = 5.94, P = 0.012) (72). Astolfi et al.’s follow-up of 105 pediatric transplant patients reported pathological fractures in 21% of children pre- or post-transplant, with elevated risk among underweight subjects, patients with reduced height Z-scores and delayed bone age. Standard calcium and vitamin D supplementation alongside nutritional support cannot fully counteract hepatic osteodystrophy-related fracture risk, mandating continuous skeletal surveillance (73). Asymptomatic fractures predominate in pediatric high-risk subgroups. Beyond conventional lateral spinal radiography, automated vertebral fracture assessment (VFA) integrated into modern DXA scanners delivers lower radiation doses. It represents a safe, efficient screening modality for moderate-to-severe pediatric spinal fractures (31). In addition, the 2019 ISCD pediatric official position statement notes that VFA shows limited reliability for isolated Genant grade-1 mild vertebral deformities; upper-thoracic segments may become unevaluable secondary to anatomical overlay, scoliosis, or motion-related artifacts (54). Conventional spinal radiography is recommended for confirmatory work-up when VFA yields equivocal findings or when identification of an isolated grade-1 deformity would alter clinical management. Notably, dedicated validation data for VFA within pediatric liver-transplant populations remain absent (54). Nevertheless, a small cross-sectional study by Tamminen et al. of 19 pediatric transplant patients argued that DXA BMD measurements fail to recapitulate intrinsic pediatric skeletal pathology fully; pediatric fracture etiology centers on disrupted bone turnover and attenuated trabecular architecture rather than isolated bone mass reduction (74).

Recent single-center retrospective evidence further explored the performance of routine contrast-enhanced abdominal CT for skeletal health screening among pediatric LT recipients (75). In a cohort including 62 pediatric transplant recipients with paired CT and DXA assessments completed within a 3-month window, sagittal-plane vertebral attenuation values exhibited moderate positive correlation with DXA-derived age-adjusted Z-scores, whereas correlation was weaker for axial-plane measurements. With an optimal sagittal-plane cut-off of 188 HU, this metric yielded moderate sensitivity and specificity for identifying patients with low age-matched bone mineral status. Of note, owing to dynamically maturing trabecular microarchitecture and open growth plates in growing children, CT-derived vertebral attenuation cannot serve as a direct substitute for DXA in pediatric populations. It should be interpreted merely as supplementary screening information when formal DXA assessment is logistically difficult to accomplish (75). Children with underlying autoimmune liver etiology tended to demonstrate poorer bone status in this pediatric cohort, which highlights the necessity of intensified skeletal surveillance for this high-risk subgroup (75).

Donor-recipient sex matching also impacts pediatric vertebral radiodensity, per a retrospective cohort by Miki et al. enrolling 116 pediatric LT recipients. Male donor-to-male recipient pairs exhibited the lowest pre-transplant vertebral HU values, whereas male donor-to-female recipients displayed the highest baseline vertebral density among four subgroups (P = 0.03). Both male donor-recipient cohorts demonstrated significantly greater post-transplant vertebral density decline compared to female donor groups (P = 0.001) (53). Overall, specialized diagnostic and therapeutic frameworks for pediatric LT-associated skeletal complications remain incomplete, with a shortage of pediatric-specific fracture prediction scoring systems and large-scale long-term clinical trial data.

## 6 Conclusion

Liver transplantation-related osteoporosis and fragility fractures develop through multi-stage, synergistic pathogenic mechanisms spanning pre- and post-transplant periods, with the first postoperative year as a critical high-risk window. Conventional DXA combined with vertebral CT attenuation values and simplified scoring systems may facilitate stratified risk screening. General calcium/vitamin D supplementation and antiresorptive agents constitute core interventions, while DKK-1/Wnt/β-catenin targeted therapy exhibits promising therapeutic potential. Pediatric recipients demand age-specific skeletal assessment standards. Current clinical surveillance and intervention rates remain suboptimal. Large prospective cohorts are needed to validate standardized peri-transplant bone health protocols and optimize individualized targeted strategies to mitigate skeletal adverse outcomes for transplant recipients.
